# Towards More Structure: Comparing TNM Staging Completeness and Processing Time of Text-Based Reports versus Fully Segmented and Annotated PET/CT Data of Non-Small-Cell Lung Cancer

**DOI:** 10.1155/2018/5693058

**Published:** 2018-11-01

**Authors:** Raphael Sexauer, Thomas Weikert, Kevin Mader, Andreas Wicki, Sabine Schädelin, Bram Stieltjes, Jens Bremerich, Gregor Sommer, Alexander W. Sauter

**Affiliations:** ^1^University Hospital Basel, University of Basel, Department of Radiology, Petersgraben 4, 4031 Basel, Switzerland; ^2^4Quant, Technoparkstrasse 1, 8005 Zurich, Switzerland; ^3^University Hospital Basel, University of Basel, Department of Oncology, Spitalstrasse 21, 4031 Basel, Switzerland; ^4^University Hospital Basel, University of Basel, Clinical Trial Unit, Department of Clinical Research, Spitalstrasse 12, 4056 Basel, Switzerland

## Abstract

Results of PET/CT examinations are communicated as text-based reports which are frequently not fully structured. Incomplete or missing staging information can be a significant source of staging and treatment errors. We compared standard text-based reports to a manual full 3D-segmentation-based approach with respect to TNM completeness and processing time. TNM information was extracted retrospectively from 395 reports. Moreover, the RIS time stamps of these reports were analyzed. 2995 lesions using a set of 41 classification labels (TNM features + location) were manually segmented on the corresponding image data. Information content and processing time of reports and segmentations were compared using descriptive statistics and modelling. The TNM/UICC stage was mentioned explicitly in only 6% (*n*=22) of the text-based reports. In 22% (*n*=86), information was incomplete, most frequently affecting T stage (19%, *n*=74), followed by N stage (6%, *n*=22) and M stage (2%, *n*=9). Full NSCLC-lesion segmentation required a median time of 13.3 min, while the median of the shortest estimator of the text-based reporting time (*R*1) was 18.1 min (*p*=0.01). Tumor stage (UICC I/II: 5.2 min, UICC III/IV: 20.3 min, *p* < 0.001), lesion size (*p* < 0.001), and lesion count (*n*=1: 4.4 min, *n*=12: 37.2 min, *p* < 0.001) correlated significantly with the segmentation time, but not with the estimators of text-based reporting time. Numerous text-based reports are lacking staging information. A segmentation-based reporting approach tailored to the staging task improves report quality with manageable processing time and helps to avoid erroneous therapy decisions based on incomplete reports. Furthermore, segmented data may be used for multimedia enhancement and automatization.

## 1. Introduction

Non-small-cell lung cancer (NSCLC) is a common malignant tumor and the leading cause of cancer-related death worldwide [1]. NSCLC is staged according to the American Joint Committee on Cancer (AJCC) and the Union for International Cancer Control (UICC) manuals that implement current medical knowledge to optimize patient survival [2]. 18F-fluorodeoxyglucose (FDG) PET/CT is currently considered the standard imaging procedure for noninvasive staging of NSCLC [3].

Accurate image-based staging is key for further diagnostic workup and therapy management. However, the discordance between preoperative staging using PET/CT and surgical pathology is considerable: according to Cerfolio and Bryant, approximately 32% of patients are preoperatively understaged [4]. Furthermore, patients with predicted stage IA have a pathological confirmation of this stage in only 65% [5]. Sources of misclassification may be biological and technical limitations [6], but the process chain from image acquisition, interpretation, and reporting may be error-prone as well [7]. This has not yet been quantified in the context of NSCLC staging. Such misclassification might be reduced by introduction of more structured text reports [8].

Next to the discordance as shortcoming of the current reading process, it can be argued that this process does not extract all potentially relevant information from imaging data. Despite being only partially reflected in the current staging system, factors like tumor burden are of great prognostic relevance for patients with NSCLC. Oh et al. have shown that, in patients with brain metastases, the overall survival is inversely correlated with the volume of all metastases [9]. Moreover, the number of positive lymph nodes has been identified as an independent prognostic factor of survival in patients with stage N1 disease. Furthermore, a recent study by He et al. pointed out that advanced NSCLC can be further divided into 3 prognostic subgroups: according to the genotype, number of metastatic organ sites, and metastasis lesions [10]. Such detailed information is neither included in regular text-based reports nor covered by structured reporting tools. Contrarily, new applications such as multimedia enhancement and image segmentation can capture this information [11]. However, in the light of health-care cost savings, personnel shortages, and subsequently decreasing available reporting time [12], investment into such new approaches requires careful consideration.

The aim of this study was to quantify the amount of TNM information missing in conventional text-based PET/CT reports for staging of NSCLC, to outline an implementation for structured, multimedia-enhanced segmentation-based reporting of imaging findings in NSCLC, and to compare this approach to conventional, text-based reporting in terms of staging accuracy and processing time.

## 2. Materials and Methods

### 2.1. Patient Population

The local ethics committee approved this retrospective, observational study. All work was conducted in accordance with the Declaration of Helsinki (1964).

**Figure 1:**
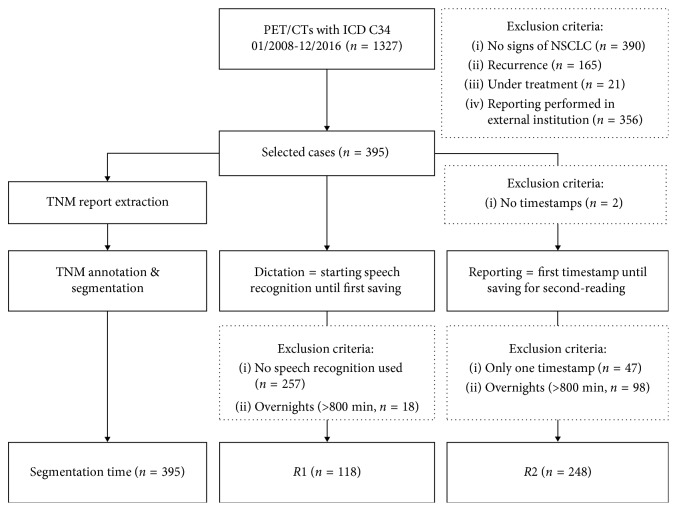
Study flowchart. 395 (30%) NSCLC patients that underwent PET/CT for primary staging were selected. These cases were included for both TNM extraction and segmentation.

From 1327 FDG-PET/CTs examinations that were performed with the ICD-10 diagnosis code C34 between 01/2008 and 12/2016, 395 were selected according to the inclusion criteria “histologically proven NSCLC” and “primary staging situation.” Exclusion criteria are listed in Figure 1.

### 2.2. Imaging Protocol and Reporting

PET/CT examinations were performed on an integrated PET/CT system with 16-slice CT (Discovery STE, GE Healthcare, Chalfont St Giles, UK) from 01/2008 to 11/2015 and on a PET/CT with 128-slice CT (Biograph mCT-X RT Pro Edition, Siemens Healthineers, Erlangen, Germany) from 12/2015 to 12/2016. Before tracer injection, patients were fasting for at least 6 h. Scans were obtained 1 h after intravenous injection of 5 MBq FDG/kg body weight at glycaemic levels below 10 mmol/L. All text-based reports were created by a resident in nuclear medicine in daily clinical practice using electronic reports with findings structured by anatomic regions [13] and were reviewed and signed by a board-certified radiologist and a board nuclear medicine physician in consensus.

### 2.3. Report-Based TNM Extraction

A dual-board-certified radiologist and nuclear medicine physician (G.S.) interpreted and extracted the TNM stage by analyzing the text-based reports for T (1–4), N (0–3), and M (0-1) descriptors or other text information that are stage defining without access to other clinical information or PET/CT images. It was also recorded whether the TNM or UICC stage was mentioned in the report explicitly. The descriptor was reported as missing when neither the TNM descriptor nor equivalent stage-defining information such as tumor size was found. From the extracted TNM, we derived the UICC (7th edition) stage.

### 2.4. TNM Annotation and Image Segmentation

For each patient, the PET/CT image dataset was loaded to a 3D Slicer-based segmentation software (version 4.6.2, BSD-style open source license, Slicer Python Interactor 2.7.11, http://www.slicer.org, Boston, USA) [14]. This software was modified in order to support direct-structured annotation using a set of labels that represent predefined features of lesions according to the TNM classification (7th edition) (Table 1). More detailed information about the subcategories can be found in the supplementary material (Tables S1–S3). Annotation and volumetric image segmentation with reference to the report was performed manually in random order by a dual-board-certified radiologist and nuclear medicine physician (A.S., reader 1, *n*=168) with 9 years' experience in PET/CT reading as well as a supervised radiology resident with 2 years of professional experience (T.W., reader 2, *n*=227). Each lesion was segmented as a 3D volume defined by multiple 2D regions of interest (ROIs) that were drawn on contiguous transversal slices of the CT component of the dataset. Fused PET information was used in addition whenever the boundaries of a lesion were not clearly definable on CT.

Output files were saved as JavaScript Object Notation (JSON) files, including time measurement registries and annotations (3007 lesions in total). From these, TNM and UICC were automatically derived.

### 2.5. Data Analysis

For comparison, we focused on TNM/UICC stage as qualitative and on time as quantitative measures.

### 2.6. TNM/UICC

The TNM information extracted from the text-based reports was analyzed for the frequency of missing information using Excel 2010 (14.0, Microsoft Corporation, Redmond, USA).

### 2.7. Estimators of Text-Based Reporting Time

The RIS timestamps were recorded since 05/2010 and registered in 393 of 395 cases. Since reporting time cannot be derived directly from RIS time entries, we used three timestamps for estimation: starting speech recognition, first saving, and saving for second reading. The consistency between RIS time entries and real-time was confirmed by testing 5 sample reports.

As a lower estimator of the text-based reporting time, we defined the time between starting speech recognition and first saving as *R*1. Cases in which no speech recognition was used (*n*=257) and registries >800 min (= overnight, *n*=18) were excluded.

As an upper estimator of the text-based reporting time, we defined the time between the start of speech recognition or first saving until the saving for second reading as *R*2. Cases without speech recognition which were only saved once (*n*=47) and registries >800 min (= overnight, *n*=98) were excluded.

To evaluate if these estimators are representative, we used all oncological PET/CTs from 05/2010 to 01/2018 (*n*=14239) (Table S4). A model based on expectation-maximization (EM) algorithm [15] was applied for outlier detection and simulation of lower (*R*1) and upper (*R*2) boundaries for verification. Using a Gaussian mixture model, we identified registries >800 min as outliers. Then, we developed a mathematical simulation using *R* (3.4.3, R Core Team, GNU GPL//RStudio, 1.1.414, RStudio Inc., Boston, USA) to differentiate interruptions from real reporting time (*R*1 and *R*2). This model was used to test the upper (*R*2) and lower estimators (*R*1). Further information including the *R* code can be found in Supplementary Materials modelling for reporting time estimation.

### 2.8. Segmentation Time

Segmentation time per lesion was extracted from the JSON file. 99.6% (2995/3007) lesions were segmented in <175 min. 12 lesions segmented in >800 min were excluded as outliers. Registries were analyzed regarding reader, lesion count, TNM, and UICC. Statistically significant impact factors of segmentation time were tested on RIS time registries for comparison.

### 2.9. Statistical Analysis

For descriptive statistics, median, arithmetic mean, and median test were used. For statistical analysis of segmentation, we pooled the data from readers 1 and 2. For outlier detection, we utilized mixture modelling with maximum likelihood estimation for RIS time registries. Spearman's rank correlation (*r*_s_) coefficient was used for ordinal (e.g., UICC with time) and Pearson's correlation coefficient (*r*) for interval-scaled data (e.g., lesion count) to evaluate correlation. For linear models, we used ANOVA (analysis of variance; *R* ^2^, *F*) to show significance. To evaluate multifactorial impact, we used automatic linear modelling in SPSS (IBM Statistics 22.0.0.0, IBM Corporation, New York, USA). To include impact factors, we used a 95% confidence level and Akaike information criterion (AIC). We used the Wilcoxon signed-rank test to compare two related samples such as dictation and segmentation time of the same patient. For differences in distribution, we used Mann–Whitney U test (*U*) for independent samples like reader dependency or incomplete versus complete reports and Kruskal–Wallis if there were more than two variables. To test normal distribution, Kolmogorov–Smirnov was used. The t-test was used to determine significant differences in normal distributed samples. *P* < 0.05 was set as the level for statistical significance.

## 3. Results

**Figure 2:**
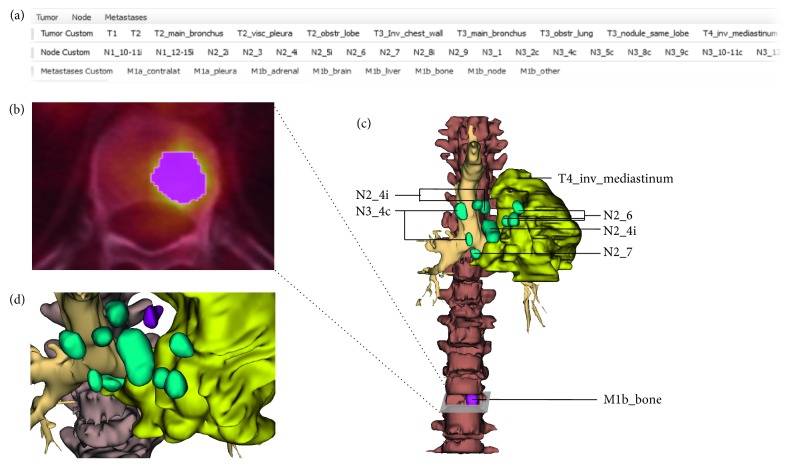
Example of a three-dimensional annotation and segmentation of NSCLC lesions from FDG-PET/CT data of a 71-year-old male patient with squamous cell carcinoma. (a) After selecting the label from the toolbar, (b) the lesions were manually segmented. (c) Tumor lesions as a visual report of primary staging including stage information and location. (d) Detailed view of the infiltrating primary tumor (yellow), lymph node metastasis (green), and pleural metastasis (purple).

**Figure 3:**
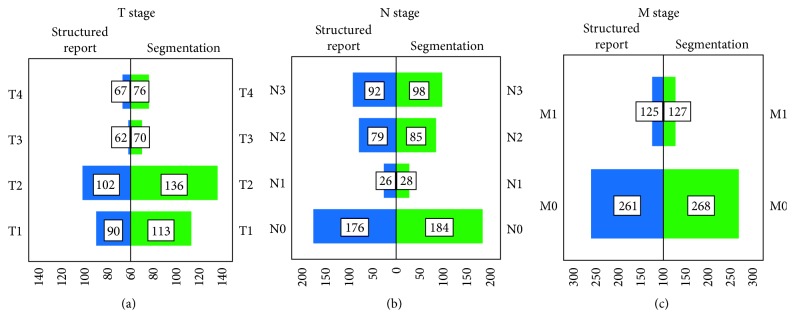
Completeness of TNM information and stage distribution. The T (a), N (b), and M (c) stages of the different TNM descriptors (7th edition), as well as their frequency in segmentation and the text-based reports, are shown.

Our NSCLC study population (*n*=395) comprised 28% female and 72% male patients with ages between 38 and 97 years (71.7 ± 10.5 years). An example of the annotation and segmentation process of NSCLC lesions is shown in Figure 2 for a 71-year-old male patient case suffering from T4 N3 M1 squamous cell carcinoma. The distribution of the T/N/M stages according to the text-based reports and segmentations is presented in Figure 3.  Table 2 gives an overview of descriptive time statistics for both segmentation and text-based reporting.

### 3.1. Completeness of TNM Information in Text-Based Reports

Due to lack of information, TNM extraction was not possible for 86 out of 395 text-based reports (22%). Of these, the T stage was most frequently affected (*n*=74, 19%) as shown in Figure 3. Stage identification information was missing in 6% for the N (*n*=22) and in 2% (*n*=9) for the M descriptor. In four cases (1%), TNM information was missing completely. An explicit mention of the absence of metastasis was present in 20% for nodal (*n*=80) and in 32% (*n*=126) for distant metastasis. A statement on the specific TNM or UICC stage was made in only 6% (*n*=22) of the text-based reports.

### 3.2. Analysis of Text-Based Reporting Time

**Figure 4:**
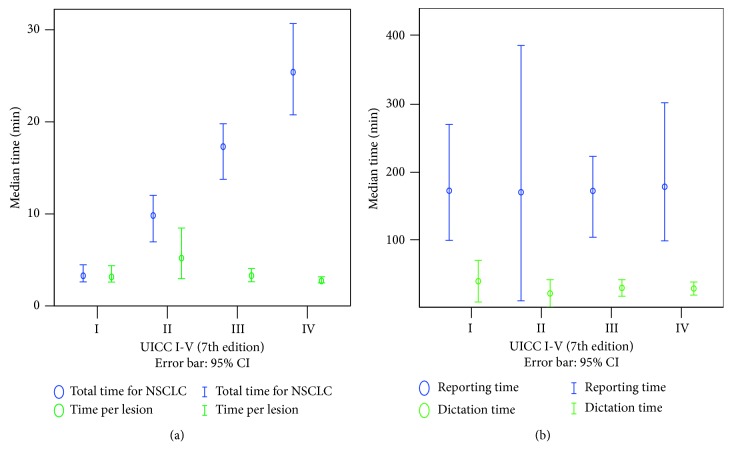
Comparison of time needed for staging depending on UICC stage. The median is indicated by a circle, accompanied by its 95% confidence interval. (a) Segmentation time is correlated with UICC stage, whereas the medians of total time and time per lesion show an inverse correlation. (b) Neither *R*2 nor *R*1 is related to the UICC stage. *R*1 = lower estimator of the text-based reporting time. *R*2 = upper estimator of the text-based reporting time.

The reporting time of the extracted RIS reports was estimated from *R*1 as the lower benchmark and *R*2 as the upper benchmark. The median total time was 18.1 min for *R*1 (*n*=118) and 151.6 min for *R*2 (*n*=248) (Table 2). To assess the general applicability of this approach, a simulation was done based on a larger number of non-disease-specific PET/CT examinations performed between 05/2010 and 01/2018 (Table S4). Here, a median of 26.6 min (*n*=3700) for *R*1 as the lower benchmark and 146.1 min for *R*2 (*n*=7190) as the upper benchmark were found (Table 2). There was no significant difference between the sampled and modeled *R*1s (*F*=10.34, *p*=0.603) but between the sampled and modeled *R*2s (*F*=25.918, *p*=0.010). UICC stage and lesion count were neither correlated with *R*1 (UICC: *r*_s_=0.002, *p*=0.986; lesion count: *r*=−0.042, *p*=0.652) nor with *R*2 (UICC: *r*_s_=0.031, *p*=0.649; lesion count: *r*=0.119, *p*=0.061) (Figure 4). Those text-based reports where report-based TNM extraction was possible due to sufficient information (78%) took longer (*R*1: 19.5 min) than text-based reports with no or incomplete TNM information (*R*1: 14.8 min).

### 3.3. Analysis of Segmentation Time

In contrast to the text-based reports, TNM and UICC could be defined readily in all cases by annotation and segmentation. Reader 1 (experienced reader, 168 cases, 1172 lesions) required a median of 13.8 min, and reader 2 (resident, 227 cases, 1835 lesions) needed a median of 17.2 min per case. The median test (*p*=0.184) showed no significant difference, even if the differences in distribution show a slightly faster segmentation by reader 1 (*U*=22113*p*=0.002). The central tendencies regarding T (*U*=0.091*p*=0.927), N (*U*=−0.881, *p*=0.378), and UICC (*U*=−1.161, *p*=0.246) stages and age (*t*=1.01, *p*=0.312) do not differ significantly between both readers. M stage shows that reader 2 (36.6%) segmented more cases with distant metastases than reader 1 (*U*=−2.1, *p*=0.035), which in part explains longer segmentation time periods. Results from both readers were used for further analysis.

**Figure 5:**
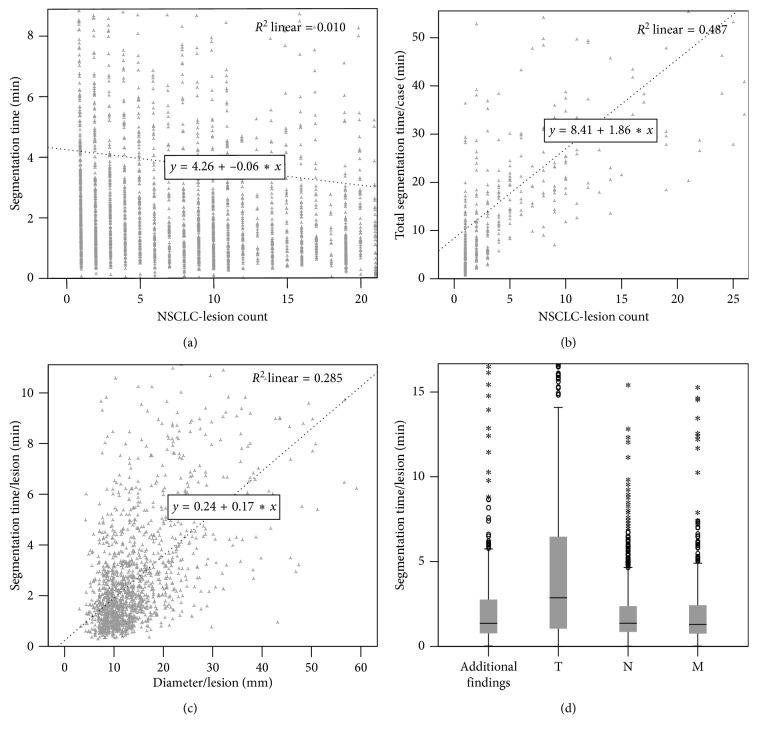
Factors influencing the segmentation time. (a) Scatter plot of NSCLC-lesion count versus segmentation time per lesion (grey): segmentation time per lesion slightly decreases with lesion count as shown by a linear regression line (black dotted). (b) Scatter plot of NSCLC-lesion count versus total segmentation time: the linear regression (black dotted) shows that total segmentation time increases with lesion count. (c) Scatter plot of lesion diameter versus segmentation time per lesion showing an increase in segmentation time with lesion diameter. (d) Box plots displaying the required segmentation time per individual lesion depending on its main category.

The segmentation required a median of 13.3 min for the staging of NSCLC and 3.8 min extra, if there were additional findings (Figure 5). For segmentation of one lesion, a median of 1.5 min was needed.

The time registries showed that segmentation-based staging was dependent on the lesion count and tumor stage. As the lesion count increased, the total segmentation time increased linearly (*R* ^2^=0.361, *F*=221.536, *p* < 0.001), whereas time per lesion slightly decreased (*R* ^2^=0.01, *F*=32.4, *p* < 0.001) (Figure 5). According to linear regression, an average of 2.1 min was needed for each additional lesion. In addition, lesion size (independent from the lesion type) showed a positive correlation with segmentation time (*R* ^2^=0.284, *F*=1106.466, *p* < 0.001).

Table 3 gives an overview of the relationship between diameter and segmentation time per lesion. The average T lesion diameter was 18.1 mm. The median time required per T lesion was 2.9 min and, according to linear regression, each additional T lesion led to an increase by 0.84 min (*F*=13.0, *p* < 0.001) on average. In contrast to the T stage (average count: 1.7), average N (average count: 2.9) and M (average count: 5.6) lesion counts were higher. On the other hand, average diameters of N (12.2 ± 4.8 mm) and M (13.0 ± 4.6 mm) lesions were smaller. Subsequently, segmentation times per metastatic and nodal lesion were approximately half of T lesions (Table 3) (T vs. N lesions: *p* < 0.001, T vs. M lesions: *p* < 0.001).

The total time for the segmentation correlated with the T (*r*_S_=0.426, *p* < 0.001), N (*r*_S_=0.694, *p* < 0.001), and M (*r*_S_=0.512, *p* < 0.001) stages and thus also with the UICC stage (*r*_S_=0.564, *p* < 0.001). N (*F*=40.9, *p* < 0.001) and M (*F*=42.5, *p* < 0.001) stages have a greater impact on total staging time as the T stage (*F*=17.0, *p* < 0.001), estimating 67.3% for N stage, 23.4% for M stage, and only 9.3% for T stage. A median of 5.1 min segmentation time was needed for UICC I/II versus 6.8 min for UICC III/IV per T stage (*U*=32355, *p* < 0.001).

In contrast to the reporting times, the median segmentation time for those cases with sufficient information for TNM extraction in the text-based reports (78%) was not longer than for those with no or incomplete TNM information in the text-based reports (13.3 min for each group).

## 4. Discussion

Our objective was to analyze the amount of TNM information missing in text-based PET/CT reports for staging of NSCLC and to compare this conventional reporting with a new segmentation and annotation approach of the total tumor burden. The most important findings can be summarized as follows: TNM stage was frequently missing in structured text-based PET/CT reports (22%). Annotated image segmentation always includes tumor stage and thus enhances the quality of the diagnosis. Segmentation time (median = 16.3 min) increases with the TNM and UICC stage as well as the lesion count, whilst text-based reporting times (lower boundary estimator *R*1 = 18.1 min) are neither correlated with the tumor stage nor lesion count.

Definitions and implementations of free text versus structured text reporting are currently under debate. According to Weiss et al., structured reporting can be divided into the following three steps [16]:

- Level 1: use of common headings
- Level 2: use of subheadings specifying organs or organ systems (“itemized”)
- Level 3: use of standardized language (“clickable”)

Most guidelines for PET/CT suggest 3 principal style formats of reporting: order of importance, anatomic site, and hybrid [13]. In our institution, the preferred style is driven by the anatomic site. In our sample, we found that in 22% of text-based level 2 structured reports the TNM stage is missing. Since further treatment depends in particular on the tumor stage, the absence of TNM in 22% of the examined cases is alarmingly high. In such cases with undocumented TNM, miscommunication and uncontrolled interpretation might entail misstaging and wrong treatment decisions. Furthermore, missing TNM will decrease efficiency of multidisciplinary tumor boards. It is noteworthy that the tumor stage is an important part of the report for the oncologist and missing findings, in general, are the most common cause of malpractice suits [17].

A first approach that might come to mind as a potential remedy is the introduction of level 3 structured reporting approaches. However, as direct links between the text and the image are missing, this approach offers limited options in terms of reporting tumor burden and communicating measurements. Therefore, according to Folio et al., the use of image-based annotated measurements in a standardized format would significantly improve the report quality even beyond the results of text-based structuring alone [18].

However, as the time available per image becomes increasingly shorter [19] and increasing workload can be a source of error on its own [20], an evaluation of the required time for segmentation is of pivotal importance. In our study, a median time of 13.3 min was needed for segmentation of the total NSCLC tumor burden with explicit annotation of T, N, and M lesions. Velazquez et al. have compared manual and semiautomatic computed tomography- (CT-) based segmentation of primary lung tumors [21]. The authors measured a mean segmentation time of 10.6 min (range: 4.85–18.25 min) for the manual slice-by-slice delineations. Furthermore, in the Multimodal Brain Tumor Image Segmentation Benchmark (BRATS), MRI scans were segmented by a trained team of radiologists using 3D slicer software, taking about 60 min per subject [22]. Thus, in this context, our segmentation times seem to be quite low in comparison.

To estimate the time required for normal text-based reporting as a reference value, we used a time stamp-based approach on the sample. Since time stamps only give a rough estimate of the true reporting time, we tried to fortify our estimate with a modeled timing based on an extensive sample of PET/CTs. While the median of *R*1 between the two groups are comparable, *R*2 of the samples differed significantly from the simulation. This suggests the lower benchmark (*R*1) to be more reliable because of the small difference between the sample and modelling. According to a web-based survey performed by Karantanis et al. [23], most PET/CT readers estimate the mean reading time between 15 and 20 min, which is comparable in particular to our lower benchmark. The duration of comparable whole-body CT reports has been calculated based on RIS entries at approximately 30 minutes [24]. This is within the range of *R*1–*R*2. Overall, based on our data, the reference values published in literature, and from our own personal experience, it seems justified to estimate the reading time for a PET/CT exam in NSCLC in between 20 and 30 minutes. Interestingly, the time requirements for conventional text-based reporting in our analysis were independent of factors such as lesion number or TNM stage.

We have analyzed factors that influence the time needed for segmentation. Here, the time required can be estimated by case complexity and is dependent on lesion number, tumor size, infiltration, and metastasis. The relevance of total tumor burden, expressed as total tumor volume or lesion count, for patient prognosis has been shown by several studies [10, 25, 26]. This is also recognized by the International Association for the Study of Lung Cancer (IASLC), who in the framework of the current 8th edition of the TNM staging system for lung cancer, gives a strong recommendation for physicians to record the number of metastatic lymph nodes (or stations) in their staging reports [25]. It follows that the process of segmentation, with the search for all lesions and definition of each single lesion extension, is the only possibility to capture the tumor burden thoroughly and relate it to prognostic factors. Next, text-based reporting is frequently only a description of the major tumor burden and will never reflect every single lesion in full extent. This emphasizes the importance to develop methods for reporting towards more dedicated tumor stage information. Furthermore, while a segmentation of raw image data is largely independent of individual interpretations or these are objectively traceable, level 1 and 2 reports are commonly misinterpreted [27]. Therefore, segmentation-based reports with supplementary interpretations would be desirable, because they could enhance objectivity in the communication of radiological findings.

Beyond that, segmentation enables a multitude of new applications. It goes without saying that these are neither limited to NSCLC as a disease entity nor to tumor staging as a diagnostic task. Full tumor segmentations may be used for staging, restaging, and follow-up assessment of various kinds of malignancies, e.g., lymphoma, breast cancer, and prostate cancer [28–30], and other fields such as pathology reporting. It can be used as enriched image-guidance to plan procedures, such as biopsies, surgical procedures [31], or radiotherapy [32]. Segmentations might also serve as training data sets for machine learning by creating a machine-readable format [33]. Additional time required for segmentation may result in time-saving in the future. Therefore, IT solutions might enhance quality of TNM staging whilst reducing the workload for radiologists.

There are some limitations in our study. Evaluation of text-based RIS reports, collection of their reporting duration, and segmentation were retrospectively performed. Therefore, there was an unavoidable selection bias. In contrast to a prospective survey of real-time reporting, it was not possible to evaluate external factors and interruptions influencing the duration of a report. Since segmentation of each case was not performed by more than one reader, inter-reader agreement cannot be evaluated. However, given the fact that text-based reports were previously performed in clinical routine and served as basis for tumor segmentation, the variability is certainly lower compared to segmentation without clinical or radiological information. Although the median segmentation time of both readers was comparable, differences in distribution were found linked to slightly different patient groups and readers' experience. In addition, segmentations were performed with a manual approach and not using semi- or automatic PET or CT segmentation that can improve the objectivity of tumor volume measurements, e.g., in head and neck cancer [34]. Furthermore, the differences in the reading environments with different sources of interruption for the reading and segmentation task complicate direct, one-to-one comparisons. Finally, the retrospective study design did also affect the validity of our data regarding the accuracy of the TNM staging information, as it was not possible to obtain clinical or even pathological confirmation for each particular lesion of interest in our rather large patient sample. In our opinion, this does not represent a major limitation in terms of the purpose of this article, as tumor stage did not serve as an endpoint of our analysis, but was investigated only with regard to its secondary effects on reporting and segmentation times.

## 5. Conclusions

In current text-based PET/CT reports, TNM staging information is frequently incomplete. Structured reporting with annotated image segmentation provides enhanced report quality with complete TNM information with manageable additional workload. Moreover, annotated image segmentation opens the door towards training artificial intelligence algorithms and better integration of imaging data in clinical workflows.

## Figures and Tables

**Table 1:** Description of label sets. The specific T-label stage is followed by a morphological descriptor that is stage defining. The N-label is defined by stage (first) and region (second) according to the IASLC lymph node map [35]. The M-label is defined by M stage and metastasis location. Additional findings that are non-NSCLC-related: T_benign referred to a benign lesion, T_other is another primary tumor, N_inflammation is an inflammatory/reactive lymph node, N_other is a nodal metastasis from another primary tumor.

T descriptor	N descriptor	M descriptor	Additional findings
T1	N1_10-11i	M1a_contralat	T_benign
T2	N1_12-15i	M1a_pleura	T_other
T2_main_bronchus	N2_2i	M1b_adrenal	N_inflammation
T2_visc_pleura	N2_3	M1b_brain	N_other
T2_obstr_lobe	N2_4i	M1b_liver	
T3_Inv_chest_wall	N2_5i	M1b_bone	
T3_main_bronchus	N2_6	M1b_node	
T3_obstr_lung	N2_7	M1b_other	
T3_nodule_same_lobe	N2_8i		
	N2_9		
T4_inv_mediastinum	N3_1		
	N3_2c		
T4_nodule_diff_lobe	N3_4c		
	N3_5c		
	N3_8c		
	N3_9c		
	N3_10-11c		
	N3_12-15c		

**Table 2:** Segmentation time versus structured reporting time.

	Segmentation time^*∗*^ (min)	Study population (NSCLC)		Simulation (miscellaneous oncological indications)	
		*R*1 (min)	*R*2 (min)	*R*1 (min)	*R*2 (min)
Mean	25.0	31.0	181.8	29.0	154.2
Standard deviation	30.9	38.2	137.2	18.7	96.5
CI	21.9–28.0	24.0–38.0	164.6–198.9	25.6–32.4	142.1–166.3
Min	0.9	1.0	3.0	0.4	0.3
Median	16.3	18.1	151.6	26.6	146.1
Max	326.0	226.0	792.9	92.9	464.4

The descriptive statistics for the collected and simulated data in minutes are shown. ^*∗*^Including additional lesions. CI = confidence interval; *R*1 = lower estimator of the text-based reporting time; *R*2 = upper estimator of the text-based reporting time.

**Table 3:** Descriptive statistics of diameter and segmentation time per lesion.

	Diameter (mm)			Time per lesion (min)		
	T	N	M	T	N	M
Mean	18.2	12.2	13.0	5.7	2.3	2.1
Standard deviation	13.7	4.8	4.6	9.7	4.9	4.8
CI	17.1–19.3	12.0–12.5	12.7–13.3	4.9–6.4	2.0–2.6	1.7–2.5
Min	4.3	4.9	4.9	0.0	0.0	0.0
Median	12.8	11.0	12.3	2.8	1.4	1.3
Max	81.0	56.6	30.6	126.0	111.0	119.2

An overview of the time required for segmentation per lesion and the lesion diameter relative to the respective T/N/M descriptors are shown. Compared to N and M lesions, T lesions have the largest diameter and highest segmentation time. CI = confidence interval.

## Data Availability

RIS time entries of all patients are provided as anonymized list in the supplementary materials (Table S4). PET/CT and corresponding annotation data are patient-related and thus confidential. Upon request, a minimal anonymized subset will be available to interested researchers. 3D Slicer and associated plugins are available in their entirety at https://github.com/Slicer/slicer.

## Supplementary Materials

Supplementary Materials Table S1: detailed description of the T-label sets. Table S2: detailed description of the N-label sets. Table S3: detailed description of the M-label sets. Table S4: list with anonymized RIS time entries. Modelling for reporting time estimation (text and R code).
