# Supplementary material for: Towards More Structure: Comparing TNM Staging Completeness and Processing Time of Text-Based Reports versus Fully Segmented and Annotated PET/CT Data of Non-Small-Cell Lung Cancer
Source: Contrast Media Mol Imaging. 2018 Nov 1;2018:5693058. doi: 10.1155/2018/5693058 (PMC6236664; doi:10.1155/2018/5693058)
Supplement: Supplementary Materials — Table S1: detailed description of the T-label sets. Table S2: detailed description of the N-label sets. Table S3: detailed description of the M-label sets. Table S4: list with anonymized RIS time entries. Modelling for reporting time estimation (text and R code). [file 5693058.f1.zip › 5693058.f1/modelling for reporting time estimation.docx]

**Modelling for reporting time estimation**

First, we minimized contextual outliers by simulating the raw data using R through exponentially distributed interruption times and normal distributed reporting times by iteration (1500, vector size (µ/sd/λ) = 100/loop). 4 independent variables were defined:

- Too short time entries due to interruptions
- Actual reporting entries (R1/R2)
- Too long time entries due to termination of the report without saving and reporting on the following day (overnights)
- time entries of unknown cause

Overnights were excluded from further modelling using the Gaussian mixture model based on the expectation-maximization algorithm (see blue line in figure 1). Since an exponential distribution for the interruptions (see green line in figure 1) and a normal distribution of the reporting time (see red line in figure 1) can be assumed, we developed a mathematical simulation based on the expectation-maximization algorithm to minimize the contextual error.

By simulating exponential and normally distributed values, lambda, mean and standard deviation were iteratively optimized. We chose 1500 iterations with a vector size of 100 for lambda, mean and standard deviation. A simulation was accepted when the mean, standard deviation, and lambda approached a specific value as the iteration increased. Finally, a good agreement between the simulated model and the raw data was reached (see orange line in figure 1).

The R code for the reporting time estimation modelling can be found in the supplementary materials.

| A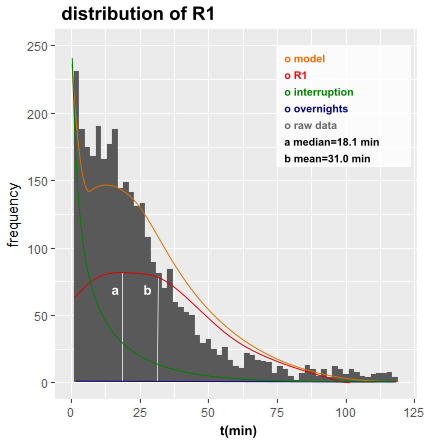 | B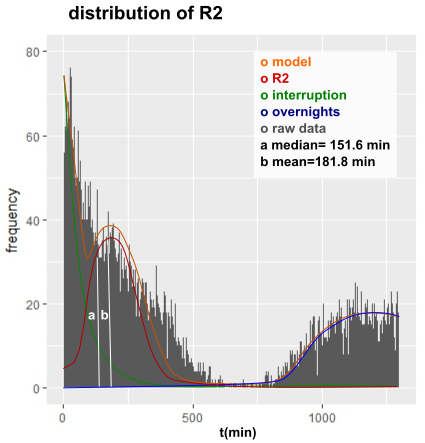 |
| --- | --- |
| Figure 1: Modelling for reporting time estimation: The raw data for R1 (A) and R2 (B) are shown as gray histogram bars. Overnights were identified and excluded using the Gaussian mixture model before the simulation. In green, the interruptions are shown as exponential function, whereas the reporting times (R1/2) were normally distributed (red). The orange line shows the simulated model that agrees well with the raw data. | |
