# Supplementary material for: Towards More Structure: Comparing TNM Staging Completeness and Processing Time of Text-Based Reports versus Fully Segmented and Annotated PET/CT Data of Non-Small-Cell Lung Cancer
Source: Contrast Media Mol Imaging. 2018 Nov 1;2018:5693058. doi: 10.1155/2018/5693058 (PMC6236664; doi:10.1155/2018/5693058)
Supplement: Supplementary Materials — Table S1: detailed description of the T-label sets. Table S2: detailed description of the N-label sets. Table S3: detailed description of the M-label sets. Table S4: list with anonymized RIS time entries. Modelling for reporting time estimation (text and R code). [file 5693058.f1.zip › 5693058.f1/TableS1-3.docx]

Supplementary Materials

| **T-Descriptor** | **Definition** |
| --- | --- |
| T1 | Tumor surrounded by lung or visceral pleura, without invasion, and more proximal than the lobar bronchus |
| T2 | Tumor surrounded by lung or visceral pleura, without invasion, and more proximal than the lobar bronchus |
| T2_main_bronchus | Involves main bronchus and is more than 2 cm distal to the carina |
| T2_visc_pleura | Invades visceral pleura |
| T2_obstr_lobe | Associated with atelectasis or obstructive pneumonitis that extends to the hilar region without involvement of the entire lung |
| T3_Inv_chest_wall | Direct invasion of the chest wall (including the superior sulcus), diaphragm, phrenic nerve, mediastinal pleura, or parietal pericardium |
| T3_main_bronchus | Involvement of the main bronchus <2 cm distal to the carina |
| T3_obstr_lung | Associated atelectasis or obstructive pneumonitis of the entire lung |
| T3_nodule_same_lobe | Tumor nodule within the same lobe as that of the primary tumor |
| T4_inv_mediastinum | Invasion of the mediastinum, heart, great vessels, trachea, recurrent laryngeal nerve, esophagus, vertebral body, or carina |
| T4_nodule_diff_lobe | Separate tumor nodule within an ipsilateral lobe |

Table S1: **Detailed description of the T-label sets.**

| **N-Descriptor** | **Definition** |
| --- | --- |
| N1_10-11i | Ipsilateral hilar node |
| N1_12-15i | Ipsilateral peripheral node |
| N2_2i | Ipsilateral superior mediastinal node S2 |
| N2_3 | Superior mediastinal node S3 |
| N2_4i | Ipsilateral superior mediastinal node S4 |
| N2_5i | Subaortic node counted as ipsilateral (left sided primary tumor) |
| N2_6 | Para-aortic node |
| N2_7 | Subcarinal node |
| N2_8i | Ipsilateral inferior mediastinal node S8 |
| N2_9 | Ipsilateral inferior mediastinal node S9 |
| N3_1 | Scalene or supraclavicular lymph nodes |
| N3_2c | Contralateral superior mediastinal node S2 |
| N3_4c | Contralateral superior mediastinal node S4 |
| N3_5c | Subaortic node counted as contralateral (right sided primary tumor) |
| N3_8c | Contralateral inferior mediastinal node S8 |
| N3_9c | Contralateral inferior mediastinal node S9 |
| N3_10-11c | Contralateral hilar node |
| N3_12-15c | Contralateral peripheral node |

Table S2: **Detailed description of the N-label sets.**

| **M-Descriptor** | **Definition** |
| --- | --- |
| M1a_contralat | Separate tumor nodule within a contralateral lobe |
| M1a_pleura | Tumor with pleural nodules |
| M1b_adrenal | Adrenal |
| M1b_brain | Brain |
| M1b_liver | Liver |
| M1b_bone | Bone |
| M1b_node | Extrathoracic lymph node |
| M1b_other | Other |

Table S3: **Detailed description of the M-label sets.**
